# Molecular subtype characteristics and development of prognostic model based on inflammation-related gene in lung adenocarcinoma

**DOI:** 10.1007/s12672-025-02513-3

**Published:** 2025-05-23

**Authors:** Xuelei Hu, Tengfei Jiang, Jinxiang Wang

**Affiliations:** 1 Department of Respiratory and Critical Care Medicine, Qilu Hospital (Qingdao), Cheeloo College of Medicine, Shandong University, Qingdao, Shandong China; 2 Department of Thoracic Surgery, Qilu Hospital (Qingdao), Cheeloo College of Medicine, Shandong University, Qingdao, Shandong China; 3 Medical Laboratory Center, Qilu Hospital (Qingdao), Cheeloo College of Medicine, Shandong University, Qingdao, Shandong China

**Keywords:** Inflammation, Lung adenocarcinoma, Prognostic value, Immune microenvironment, MMP14

## Abstract

As one of the leading causes of death worldwide, lung adenocarcinoma (LUAD) currently lacks satisfactory treatment outcomes. The inflammatory process, closely associated with the formation of the tumor microenvironment and immune evasion, plays a crucial role in LUAD development. This study utilized data from public databases to analyze inflammation-related genes (INF) associated with prognosis in LUAD. Based on differentially expressed INF, molecular subtypes of LUAD were identified. Subsequently, a novel INF scoring system was developed to establish a prognostic model for LUAD patients, assessing its independence and reliability. Comprehensive evaluations, including immune microenvironment infiltration features, somatic mutation characteristics, and differences in immune therapy responsiveness, were conducted to characterize the prognostic model associated with INF. We further selected MMP14 from the screened INF targets for further in vitro experiments. Experiments such as western blot, qRT-PCR, colony-forming assay and Transwell assay confirmed that downregulation of MMP14 could inhibit the cloning, proliferation and invasion of lung cancer cells, thus confirming the results of bioinformatics. Our findings provide evidence from a new perspective on the role of inflammation in LUAD and offer new insights for clinical precision and personalized therapy.

**Supplementary Information:**

The online version contains supplementary material available at 10.1007/s12672-025-02513-3.

## Introduction

As a lung cancer originating in the cells of the lung, lung adenocarcinoma (LUAD) is one of the leading causes of death worldwide and is of great concern [1]. Early detection and prompt medical attention are key to successful treatment of LUAD [2]. Treatment options may include surgical removal of cancerous tissue, chemotherapy, radiation therapy, immunotherapy and targeted therapy, depending on the stage and severity of the disease [3]. However, the current efficacy of LUAD treatment is not satisfactory.

Inflammation is a biological response typically triggered by damage to tissues or stimulating factors, serving as a protective reaction of the body. As part of the immune system, inflammation aims to eliminate damaged tissue or clear factors causing injury, thereby promoting the repair process [4]. However, cancer cells, along with stromal and inflammatory cells in the surrounding microenvironment, interact to form an inflammation-related tumor milieu, which drives tumor initiation, growth, progression, and metastasis [5]. The inflammation-related tumor microenvironment (TME) participates in the occurrence, development, and metastasis of lung cancer through mechanisms such as immune escape, tumor angiogenesis, epithelial-mesenchymal transition, and cell apoptosis [6]. Additionally, inflammation contributes to tumor progression and development by supplying pro-tumorigenic components to the TME [7]. In LUAD, inflammation similarly plays a crucial role in TME, and anti-inflammatory agents provide another therapeutic option for LUAD treatment [8]. Therefore, defining molecular targets that regulate tumor-promoting inflammation and deepening the understanding of the mechanisms involved in the inflammatory process are essential for further development of LUAD anticancer therapies [5].

Inflammation is a critical process in immunotherapy, which holds promising advancements in clinical practice [9]. Immunotherapy typically targets tumor cells by activating the patient’s immune system, leading to an inflammatory response in TME [10]. This treatment-induced inflammatory response is considered one of the indicators of treatment success, indicating that the immune system is launching an attack on the tumor [11]. Immunotherapy-based combination therapies show promise in overcoming resistance and improving prognosis in LUAD [12]. However, tumor immunotherapy still faces challenges such as toxicity control and low response rate [13]. Given these associations, a better understanding of the biological links between inflammation process and LUAD will help identify new therapeutic targets, enhance our understanding of the molecular mechanisms of immunotherapy resistance, and facilitate the development of more effective immunotherapy strategies.

In this study, we analyzed prognostic inflammatory associated genes (INF) in LUAD and identified INF-associated LUAD subtypes using data from public databases. We then developed the INF scoring system, developed a prognostic model for LUAD patients, and comprehensively evaluated its characteristics. We further selected MMP14 from the screened INF targets for further in vitro experiments. The in vitro results confirmed downregulation of MMP14 could inhibit the cloning, proliferation and invasion of lung cancer cells, thus confirming the results of bioinformatics. Our results provide evidence for the role of inflammation in LUAD from a new perspective, and provide new ideas for precise and individualized clinical treatment.

## Materials and methods

### Acquisition and preprocessing of LUAD sample gene transcriptome data

Using “lung adenocarcinoma” as a keyword, we downloaded transcriptome data and clinical baseline information for LUAD from two independent publicly available databases. From the TCGA database, after excluding samples without clinical survival information, we included a total of 60 normal samples and 507 LUAD samples for subsequent analysis. Additionally, based on the GEO database, we collected the dataset GSE72094, which includes LUAD sample transcriptomic matrices and clinical baseline information (Platforms: GPL15048 Rosetta/Merck Human RSTA Custom Affymetrix 2.0 microarray [HuRSTA_2a520709.CDF]). After excluding LUAD samples lacking clinical prognosis time and status, we extracted 397 samples from the GSE72094 dataset for further analysis. Similarly, based on the GEO database, we collected the GSE31210 (n = 106) (Platforms: GPL570[HG-U133_Plus_2] Affymetrix Human Genome U133 Plus 2.0 Array) and GSE37745 (n = 226) (Platforms: GPL570[HG-U133_Plus_2] Affymetrix Human Genome U133 Plus 2.0 Array) datasets as independent external validation cohorts. After excluding samples lacking clinical baseline characteristics, we gathered a total of 332 LUAD samples for subsequent independent validation analysis. In the Perl programming environment, we annotated gene labels based on the platform annotation files for the two datasets. Gene labels in the TCGA database were annotated using Ensembl’s human genome browser GRCh38.p13 annotation file within Perl scripts, and gene labels with identical annotations were averaged. In the R environment, the TCGA-LUAD dataset was transformed from Count format to TPM format using the “limma” R script, and batch effects were corrected and normalized across both TCGA-LUAD and GSE72094 datasets using the “sva” R script [14, 15]. Use the “ggplot2” R package to plot principal component analysis (PCA) patterns before and after batch effect removal, in order to assess the effectiveness of data correction. Additionally, copy number variation (CNV) raw data for LUAD samples were obtained from the UCSC Xena database.

### Identification of inflammation-related gene (INF) signature and molecular subtype characterization

Using the MSigDB database, we searched for INF gene sets using “inflammation response” as a keyword and collected a total of 200 INF signatures for subsequent analysis. Differential expression analysis of INF (DE-INF) signatures was conducted using the “limma” R script, with thresholds set at |fold change|> 2 and p < 0.05. The “RCircos” R script was utilized to analyze the co-localization of DE-INF on chromosomes. In Perl, we extracted CNV frequencies of DE-INF and performed visual analysis. Consensus clustering analysis using the “ConsensusClusterPlus” R script assessed INF molecular subtype features in LUAD samples. Employing the K-means algorithm with a maximum cluster (k) of 9, clustering matrices were computed for k = 2–9, evaluating cumulative distribution function (CDF) plots and Delta Area Plots. Using the “ggplot2” R script, unsupervised principal component analysis explored the distribution patterns of INF molecular subtypes. The “survival” R script conducted log-rank analysis to assess clinical prognosis of INF molecular subtypes. 

### Analysis of potential regulatory mechanisms of INF molecular subtypes

Using the KEGG pathway reference gene set “c2.cp.kegg.v7.2.symbols”, the “GSVA” R script evaluated differential regulation of KEGG signaling pathways between INF molecular subtypes. Thresholds were set at |fold change|> 2 and p < 0.05, and differential expression gene signatures between INF molecular subtypes were identified using the “limma” R package. The “clusterProfiler” R script conducted GO and KEGG enrichment analyses on differential genes.

### Assessment of immune microenvironment infiltration characteristics

Based on transcriptome features of LUAD samples, the “estimate” R script evaluated immune infiltration status, quantitatively analyzing immune scores, stromal scores, tumor purity, and ESTIMATE scores. Using the “GSVA” R script with known marker genes of 23 immune infiltrating cells, ssGSEA algorithm quantitatively analyzed relative infiltration proportions of these cells in LUAD samples. Pearson correlation algorithm in “ggplot2” R script assessed correlations between variables.

### Construction of INF prognostic scoring system based on prognostic features

Integrating INF expression features and clinical survival information in LUAD, single-factor Cox analysis was conducted using the “survival” R script to determine INF prognostic variables. The LASSO function model was constructed using the “glmnet” R package to identify prognostic variables of INF features. Furthermore, multi-factor Cox analysis identified independent prognostic variables of INF, calculating risk coefficients for each variable. INF scores for each LUAD sample were evaluated based on risk coefficients and expression features using the formula: ${\text{INF score}}\, = \,{\text{MMP14}}\, \times \,0.{197}\, + \,{\text{MET}}\, \times \,0.0{759}\, + \,{\text{IL7Rx}} - 0.{129}\, + \,{\text{PCDH7}}\, \times \,0.{175}\, + \,{\text{MEP1Ax}} - 0.{232}\, + \,{\text{OLR1x}} - 0.{169}\, + \,{\text{NMUR1x}} - 0.{231}$. Using the “caret” R script, LUAD samples were divided into complete INF score queues, training set INF queues, and validation set INF score queues in a 7:3 ratio. Based on optimal cutoff values of clinical survival curves, LUAD samples in three independent queues were divided into high and low INF score subgroups. In addition, in the independent external cohort (GSE31210 and GSE37745), we classified LUAD into high and low INF score subgroups based on the optimal cutoff value determined from the clinical prognostic curve. The “ggalluvial” R script plotted Sankey diagrams to explore potential associations between INF molecular subtypes, INF scores, and clinical survival status. Using the “survivalROC” R script, time-dependent ROC curves for 1-year, 3-year, and 5-year periods of LUAD samples were plotted, and corresponding AUC values were calculated.

### Independent prognostic analysis of INF scoring system and construction of nomogram diagnostic model

Combining clinical pathological features of LUAD samples, the “limma” and “pheatmap” R scripts analyzed distribution of INF scores across different clinical pathological features. Using the “survival” R script, clinical survival curves were plotted for each clinical pathological subgroup. Single-factor and multi-factor Cox analyses were conducted using the “survival” R script to assess hazard ratios (HR) and evaluate independent prognostic value of clinical pathological variables and INF scoring system. Using clinical pathological variables and INF scoring system, the “rms” R script constructed a nomogram diagnostic model to predict survival probabilities of LUAD samples for 1-year, 3-year, and 5-year periods. Calibration curves were plotted using the “regplot” R script to assess accuracy of the nomogram diagnostic model.

### Assessment of somatic mutation characteristics, drug sensitivity, and immune therapy response

Somatic mutation data (MAF) of LUAD samples were obtained from the TCGA database and preprocessed using Perl scripts. The “maftools” R script analyzed somatic mutation characteristics of INF score subgroups and calculated mutation rates for each gene. Immunotherapy response data for LUAD samples receiving PD-1 and CTLA4 were collected from The Cancer Immunome Atlas (TCIA) database and analyzed using the “ggplot2” R script to assess IPS scores of INF score subgroups. Additionally, using the Genomics of Drug Sensitivity in Cancer (GDSC) database, potential responses of INF score subgroups to small molecule targeted drugs were predicted using the “pRRophetic” R script.

### Cell culture and treatment

Human NSCLC cell lines A549 and human normal bronchial epithelial cell line BEAS-2B were purchased from the Cell Bank of the Chinese Academy of Sciences (Shanghai, China). Cells were cultured at 37 °C in a humidified atmosphere with 5% CO_2_ in RPMI-1640 medium supplemented with 10% fetal bovine serum, 100 U/mL penicillin, and 100 mg/mL streptomycin.

siRNA transfection was performed using Lipofectamine siRNA Transfection Reagent. A549 Cells were plated 24 h in six-well plates at 2 × 10^5^ cells per well with DMEM containing 10% FBS. The scrambled control or MMP14 siRNA duplexes were incubated with 5 µL of siRNA transfection reagent for 5 min at 37 °C, then the mixture was added to cell culture plate. For experiments, cells were transfected with siRNA for 48 h.

### Western blotting

Cell proteins were extracted using RIPA lysis buffer. BCA method was used to determined protein concentration. Then, the proteins were subjected to 10% SDS-PAGE electrophoresed gel, followed by electrotransferring to PVDF membranes, blocking using 5% skimmed milk at room temperature for 2 h. Next, the membranes were probed with rabbit anti-human MMP14 antibody (dilution, 1:1000; cat. No. ab53712; Abcam, Cambridge, UK) overnight at 4 °C. GAPDH (dilution, 1:10000; cat. No. ab181602; Abcam, Cambridge, UK) worked as an endogenous control. Subsequently, incubated with secondary antibody Goat Anti-Rabbit IgG (dilution, 1:20000) at 37 °C for 1 h. The bands were detected by enhanced a chemiluminescence (ECL) detection kit (Thermo Fisher Scientific, Barrington, IL, USA).

### Quantitative RT-PCR (qRT-PCR) analysis

Cells were lysed by using Trizol reagent (Invitrogen). RNA was extracted using a PureLink RNA minikit (Invitrogen). Then LifeTech high-capacity cDNA reverse transcription kit (Applied Biosystems) was used for cDNA synthesis with 1 μg of RNA, followed by qRT-PCR as per the manufacturer’s instructions. Data were analyzed according to the ΔΔCt method using GAPDH for normalization. The MMP14 sequences are listed as follows: forward primer: CAGGACAGGAACCAGTAGTAGA; reverse primer: CAGAACGACAGAAGGAAGATGG.

### Colony formation assays

A549 cells were transfected with siMMP14. Then, the transfected cells were plated in 6-well plate at 5 × 10^2^ cells per well. The medium changed every three days. The colonies were fixed in 4% paraformaldehyde for 24 h at 4 °C after 14 days. Then stained with 0.5% crystal violet in 20% methanol. Eclipse TS100 microscope was used to photograph the colonies.

### Cell viability

Cell viability was determined by MTT assay. After siRNA treatment, A549 cells were incubated with MTT (5 mg/mL) at 37 °C for 4 h. Then the cells were incubated with dimethyl sulfoxide. The optical density was read at 450 nm.

### Cell invasion assay

24-well Transwell chambers containing 8-mm pore diameter polycarbonate membrane were used for the cell invasion assay. Cell suspension containing 4 × 10^4^ cells were added into the upper chamber, while RPMI-1640 medium containing 10% FBS was added into the lower chamber. Then incubated at 37 °C with 5% CO_2_ for 24 h. The migrated cells were fixed with 100% methanol, and stained with 0.5% Crystal Violet. The invaded cell number was counted under the Eclipse TS100 microscope.

### Data analysis

All data preprocessing and analyses in this study were performed using R software (version 4.1.0) and Perl programming language. Pearson correlation algorithm was used to compute correlations between variables. Wilcoxon rank-sum test was employed for statistical differences between two groups, while ANOVA test was used for multi-group statistical analysis. Statistical differences in this study were corrected, and *p* < 0.05 was considered statistically significant to minimize false positives.

## Results

### Differential expression analysis and CNV frequency analysis of INF-related genes in LUAD

**Fig. 1:**
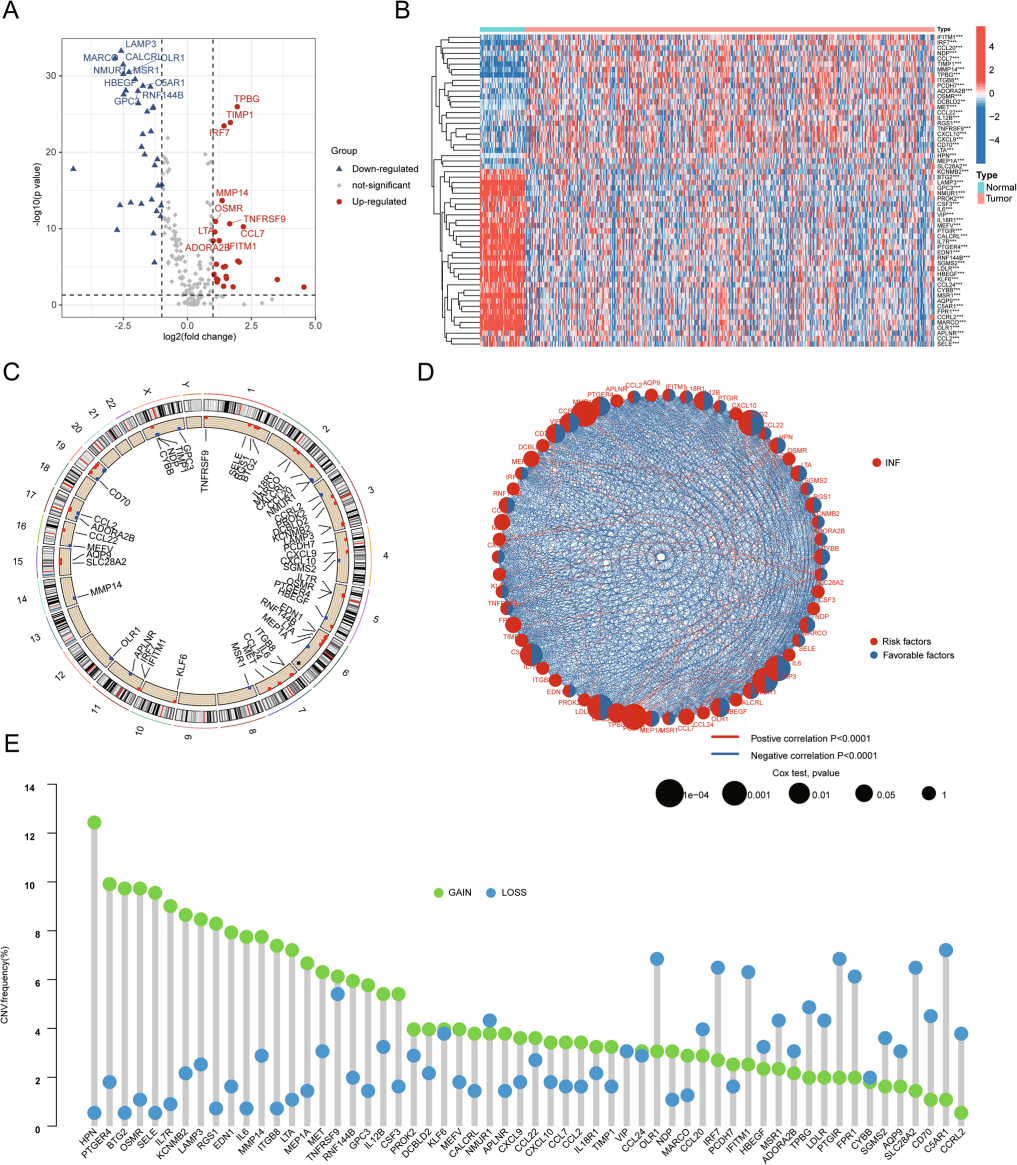
Differential expression analysis and copy number variation of INF signatures in LUAD. **A** Differential expression analysis of INF signatures between normal and tumor tissues. Differential threshold set at |fold change|> 2 and p < 0.05. Red indicates upregulation in tumor tissues, blue indicates downregulation. **B** Heatmap visualization analysis of differentially expressed INF signatures between normal and LUAD groups. **C** Co-localization analysis of DE-INF on chromosomes. **D** Network analysis of DE-INF related to prognosis. **E** Assessment of copy number variation frequencies of DE-INF

Utilizing the MSigDB database, we collected a total of 200 INF-related genes and analyzed their potential regulatory roles in LUAD development. By setting the differential threshold at |fold change|> 2 and* p* < 0.05, we assessed the differential expression of these 200 INF genes between normal and LUAD groups, as depicted in Fig. 1A. Based on this analysis, we identified 58 INF signatures that showed differential expression between the normal and LUAD groups (Fig. 1B). A chromosome co-localization plot illustrated the distribution of differentially expressed INF genes across different chromosomes, suggesting their potential association with genetic information in LUAD (Fig. 1C). Integrating clinical prognosis information of LUAD, we evaluated the potential associations of these 58 INF genes with LUAD prognosis. Network analysis indicated that 25 INF genes were potentially associated with LUAD prognosis, comprising 18 protective factors and 7 risk factors (Fig. 1D). Copy number variation (CNV) analysis revealed significant variations in CNV frequencies of INF signatures in LUAD. Genes such as HPN, PTGER4, BTG2, OSMR, SELE, and IL7R exhibited notable copy number amplifications, whereas OLR1, IRF7, IFITM1, PTGIR, and FPR1 showed significant copy number deletions (Fig. 1E). These findings highlight distinct patterns of expression and CNV variations of INF genes in LUAD, which are also associated with clinical survival prognosis. This initial characterization sheds light on the potential roles of INF in LUAD.

### Identification of molecular subtypes based on DE-INF in LUAD

**Fig. 2:**
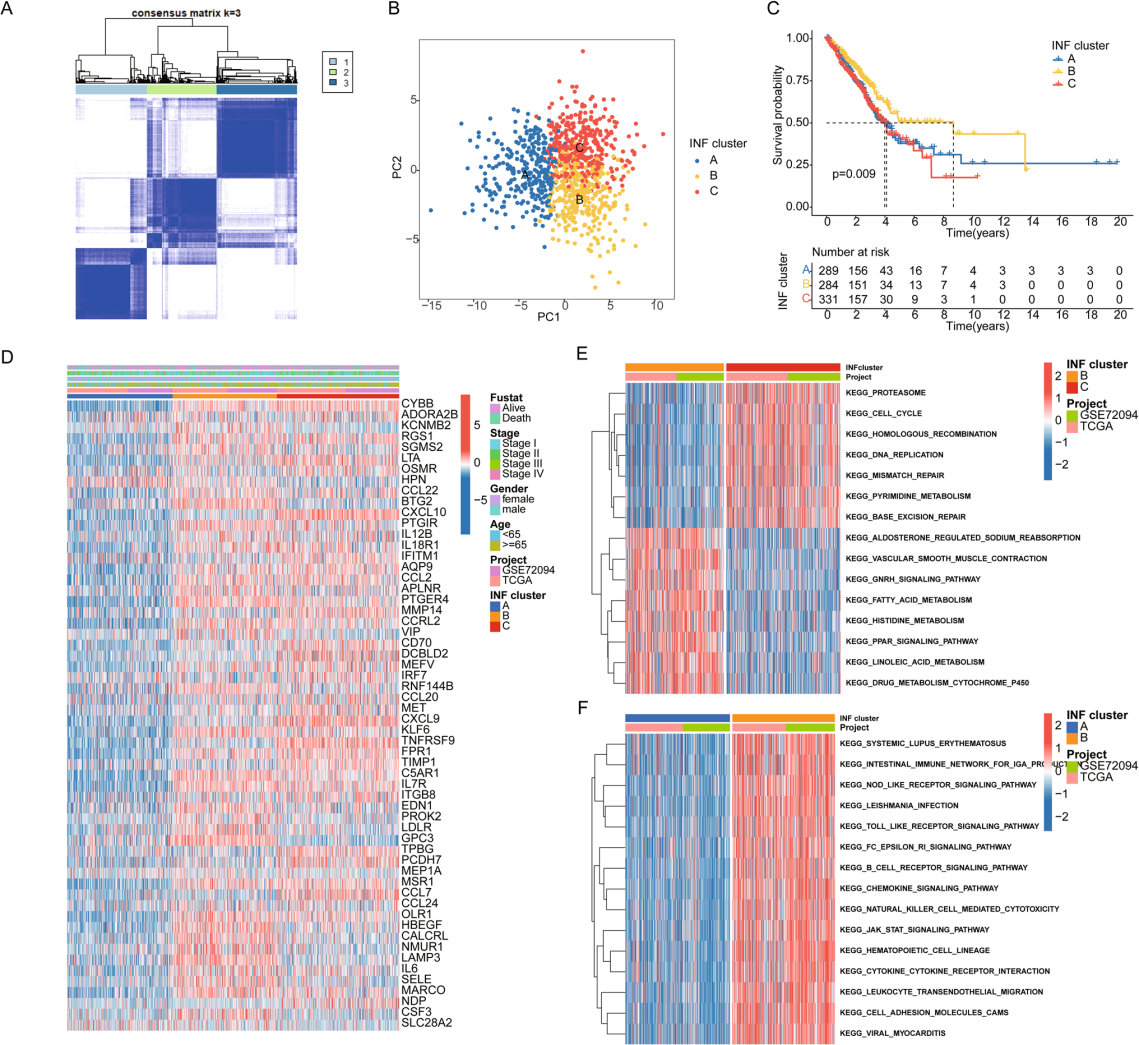
Identification of INF-based molecular subtypes in LUAD. **A** Unsupervised consensus clustering analysis of INF molecular subtypes based on INF signature expression features. Classification ratio (k) = 3. **B** PCA plot analysis of INF molecular subtype features. **C** Clinical survival prognosis curve analysis. **D** Expression profiles of INF signatures across different clinical pathological features and molecular subtypes. **E**, **F** Differential analysis of KEGG signaling pathways among INF molecular subtypes

To better understand the molecular mechanisms of INF in LUAD, we explored INF molecular subtype characteristics based on DE-INF expression features. Integrating TCGA and GSE72094 datasets, we collected a total of 904 LUAD samples to assess INF molecular subtype features (Supplementary Fig. 1A, B). Using optimal model parameters and classification ratios from consensus clustering algorithm, we identified three significantly distinct INF molecular subtype patterns in LUAD (Fig. 2A). Principal component analysis (PCA) plot results demonstrated significant separation among the three INF molecular subtypes in LUAD, indicating accurate classification of LUAD samples based on INF signatures (Fig. 2B). Clinical survival curve results showed that INF subtype B exhibited significantly better clinical survival prognosis compared to INF subtypes A and C (Fig. 2C, p = 0.009). Additionally, we evaluated the expression of 58 INF signatures across different INF molecular subtypes and clinical pathological features. The heatmap visualization revealed significantly decreased expression of most INF signatures in INF subtype A (Fig. 2D). Using GSVA algorithm, we calculated differential regulation of KEGG signaling pathways between INF molecular subtypes to elucidate potential molecular mechanisms contributing to clinical prognosis differences among INF molecular subtypes. In INF subtype C, we found significant upregulation of tumor genetic-related signaling pathways such as cell cycle, DNA replication, and homologous-recombination (Fig. 2E). Notably, in INF subtype B with the best prognosis, we identified significant upregulation of several immune modulation-related signaling pathways, including nod-like receptor signaling pathway, toll-like receptor signaling pathway, B cell receptor signaling pathway, and natural killer cell mediated cytotoxicity. This suggests that activation of immune signaling pathways may contribute to better clinical survival outcomes in LUAD (Fig. 2F).

### Evaluation of immune microenvironment infiltration features and prediction of immune therapy response in INF molecular subtypes

**Fig. 3:**
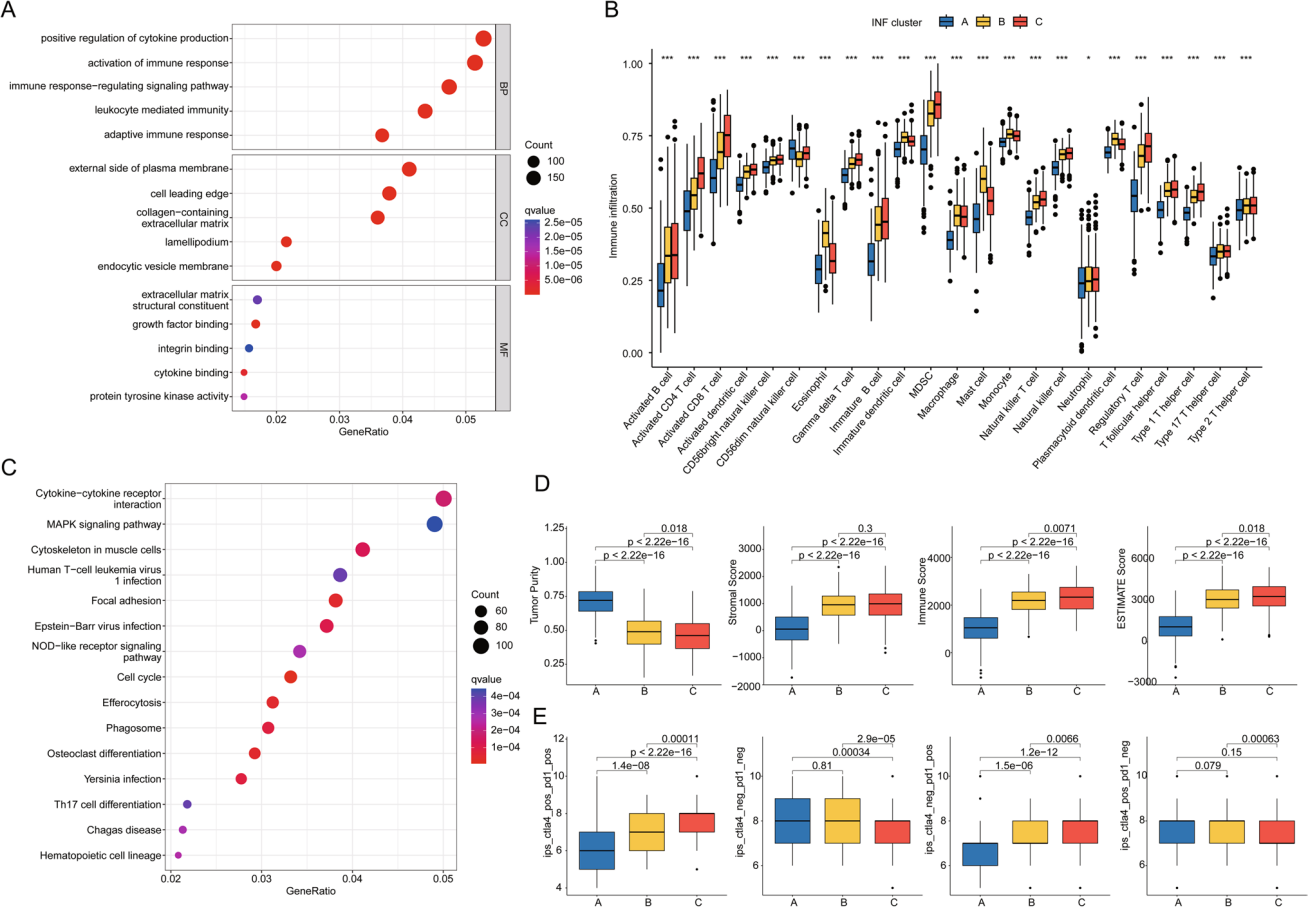
Evaluation of immune infiltration characteristics and potential molecular mechanisms of INF molecular subtypes. **A** GO enrichment analysis of genes differentially expressed between INF molecular subtypes. **B** Quantitative analysis of 23 immune infiltrating cell types between INF molecular subtypes. **C** KEGG enrichment analysis of genes differentially expressed between INF molecular subtypes. **D** ESTIMATE evaluation reveals immune infiltration status of INF molecular subtypes. **E** IPS score estimation

To further analyze key mechanisms contributing to clinical prognosis differences among LUAD molecular subtypes, we assessed differentially expressed gene signatures between INF molecular subtypes. GO analysis results indicated that differentially expressed genes between INF molecular subtypes are involved in various immune regulatory functions such as positive regulation of cytokine production, activation of immune response, and immune response-regulating signaling pathways. KEGG analysis suggested that pathways including cytokine-cytokine receptor interaction, MAPK signaling pathway, cytoskeleton in muscle cells, and human T-cell leukemia virus 1 infection may be critical mechanisms underlying clinical prognosis differences in LUAD (Fig. 3A–C). Using the ssGSEA evaluation algorithm, we quantitatively analyzed the infiltration proportions of immune cells in different INF molecular subtypes of LUAD. Results showed that in INF subtype A, the proportion of immune infiltrating cells was significantly decreased (Fig. 3B). ESTIMATE evaluation results indicated that tumor purity was significantly higher in INF subtype A compared to INF subtypes B and C, while stromal score, immune score, and ESTIMATE score were significantly lower in INF subtypes B and C (Fig. 3D). Based on the differential immune infiltration status among INF molecular subtypes, we further evaluated the response to PD-1 and CTLA4 immune therapies in INF molecular subtypes. IPS evaluation results demonstrated that INF subtype C, which had the best prognosis, exhibited the highest response to CTLA4 or PD-1 immune therapy (Fig. 3E).

### Development of prognostic model based on INF score in LUAD

**Fig. 4:**
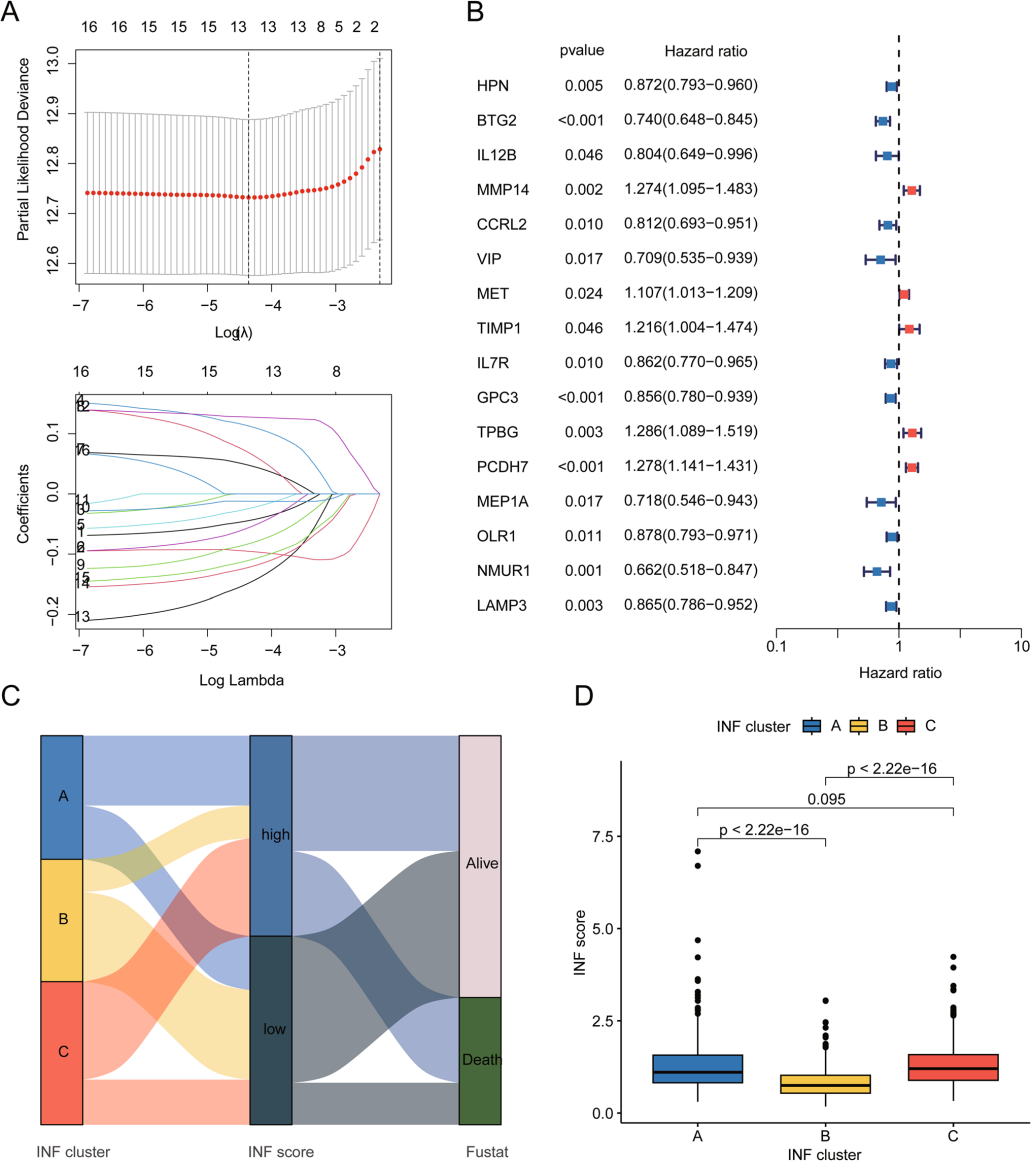
Development of prognostic model based on INF score. **A**, **B** LASSO—univariate Cox analysis identifies prognostic INF variables. **C** Sankey diagram reveals potential associations among INF molecular subtypes, INF score, and LUAD prognosis outcomes. **D** Differential analysis of INF scores

Based on INF signature expression profiles and clinical baseline data of LUAD, we developed a novel INF scoring system to stratify LUAD samples into different risk categories. Using univariate Cox analysis algorithm, we identified 16 INF gene signatures associated with clinical prognosis of LUAD, including 5 risk factors and 11 beneficial factors. Using the LASSO algorithm, we selected 13 INF features as prognostic variables and further evaluated the independent prognostic significance of each variable using multivariate Cox analysis (Fig. 4A, B). Based on the multivariate Cox analysis, we calculated an INF score for each LUAD sample (Supplementary Table 1). Sankey diagram results revealed potential associations among INF molecular subtypes, INF scoring system, and LUAD survival prognosis, suggesting that the better prognosis INF subtype B tended to have lower INF scores and better clinical outcomes (Fig. 4C). Quantitative results of INF scoring showed that in the best prognosis INF subtype B, the INF scores of LUAD samples were significantly lower compared to INF subtypes A and C, indicating that higher INF scores were associated with poorer prognosis in LUAD (Fig. 4D).

### Risk stratification based on INF score system

**Fig. 5:**
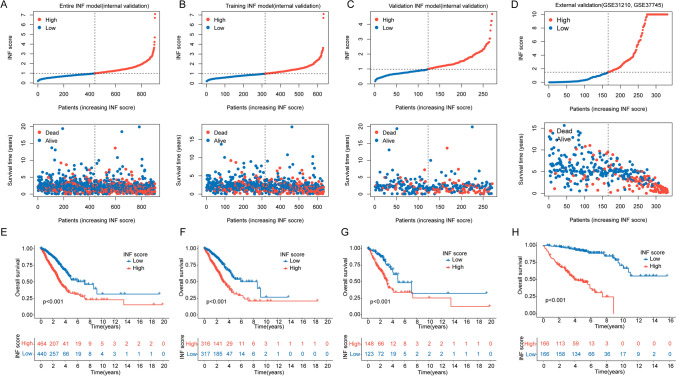
Prediction of risk stratification and prognosis analysis of LUAD samples using INF score system. **A**–**C** Subgroup division based on INF score in the complete INF score model, training set INF score model, and validation set INF score model. **D** Classification of INF score subgroups in the independent external cohort. **E**–**G** Clinical prognosis curve analysis in the three INF score models. **H** Clinical prognosis curve analysis of INF score subgroups in the independent external cohort

Based on independent prognostic variables of INF, we randomly divided 904 LUAD samples into training set, validation set, and complete set in a 7:3 ratio to verify the independence and accuracy of the INF score system in predicting risk stratification. Using optimal cutoff values for clinical survival, LUAD samples were stratified into low INF score subgroup and high INF score subgroup. As shown in Fig. 5A–C, scatter plot results indicate a close association between higher INF scores and poorer prognosis in LUAD. Additionally, by integrating the GSE31210 (n = 106) and GSE37745 (n = 226) datasets, we obtained a total of 332 LUAD samples as an independent external validation cohort (Supplementary Fig. 2A, B). Based on INF prognostic variables, we constructed an INF scoring system and performed risk stratification of the samples (Fig. 5D). The clinical prognosis curve results demonstrate that in the complete INF score model, the training set INF score model, and the validation set INF score model, LUAD samples in the low INF score subgroup exhibit significantly higher clinical overall survival rates compared to those in the high INF score subgroup. This indicates that the INF score model accurately distinguishes risk stratification in LUAD samples and reflects clinical prognosis in different subgroups (Fig. 5E–G). Notably, in the independent cohort, we observed that the OS rate of LUAD samples in the low INF score subgroup was significantly better than that in the high INF score subgroup (Fig. 5H). Based on these results, we conclude that the INF scoring system provides satisfactory consistency and reliability in predicting clinical survival outcomes in LUAD.

### Association analysis of INF score system with clinical-pathological features

**Fig. 6:**
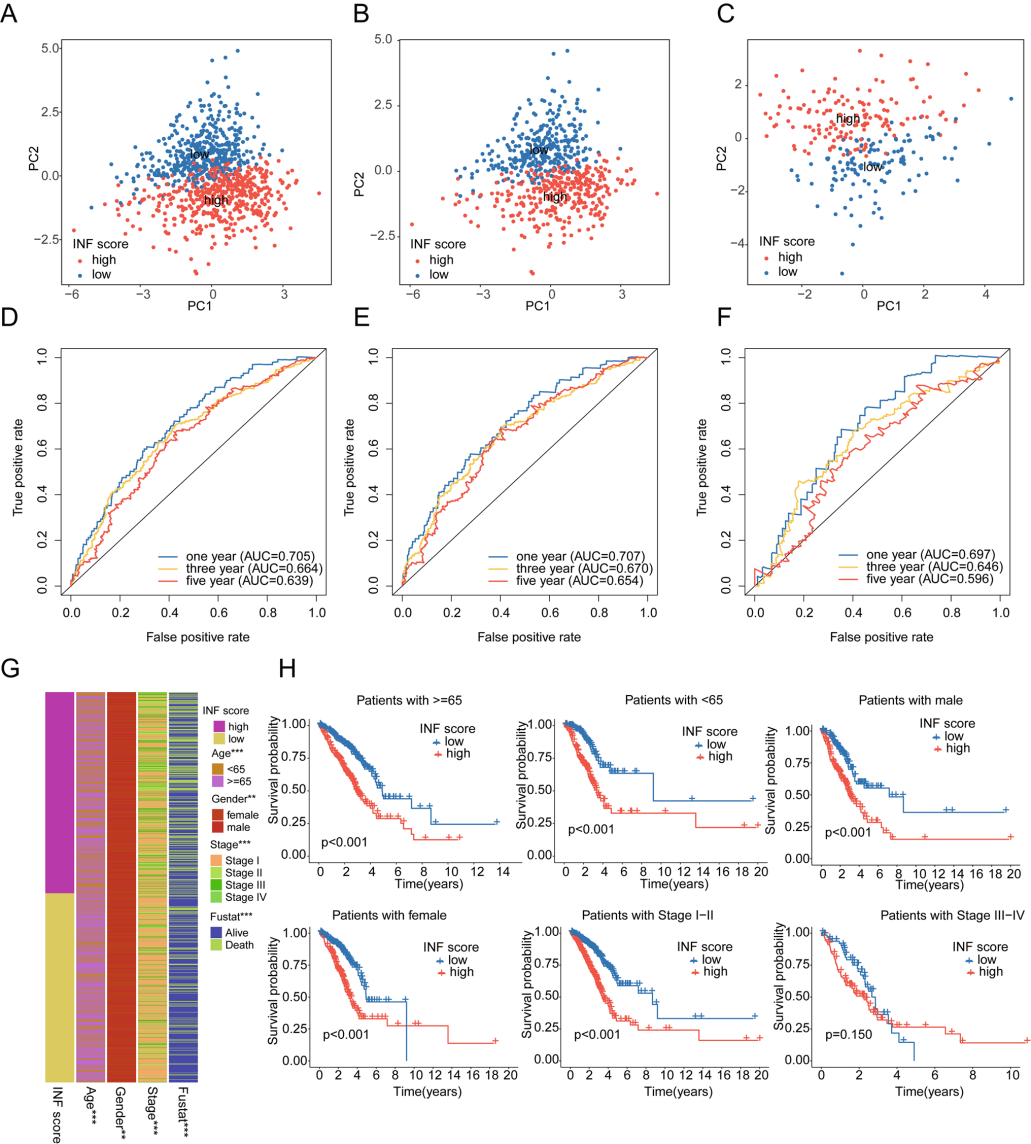
Association analysis between the INF scoring system model and clinical-pathological features of LUAD. **A**–**C** PCA plot analysis based on the INF scoring system model. **D**–**F** Time-dependent ROC curve analysis of the INF scoring system model. **G** Distribution of INF scores among different clinical-pathological features. **H** Prognostic analysis stratified by INF scoring system based on different clinical-pathological features

Subsequently, we investigated the potential associations between INF score system and clinical-pathological features of LUAD. PCA model analysis based on INF score system results indicated significant separation of low INF score subgroup and high INF score subgroup in the complete INF score model, training set INF score model, and validation set INF score model, demonstrating significant independence among INF score subgroups (Fig. 6A–C). Time-dependent ROC curve results showed AUCs of 0.705, 0.664, and 0.639 for 1-year, 3-year, and 5-year survival, respectively, in the complete INF score model, 0.707, 0.670, and 0.654 in the training set INF score model, and 0.697, 0.646, and 0.596 in the validation set (Fig. 6D–F). Furthermore, we evaluated the distribution of INF scores across different pathological features of LUAD, revealing significant differences in age, gender, stage, and survival status (Fig. 6G). Within clinical subgroups based on INF score, we found that in age, gender, and stage categories, LUAD samples in the low INF score subgroup exhibited significantly better clinical survival outcomes compared to the high INF score subgroup. Notably, there was no difference in clinical outcomes between INF score subgroups in stage III-IV (Fig. 6H). Based on these results, we conclude that the INF score model can independently predict clinical prognosis of LUAD beyond other clinical-pathological features.

### Independent prognostic analysis of the INF scoring system model

**Fig. 7:**
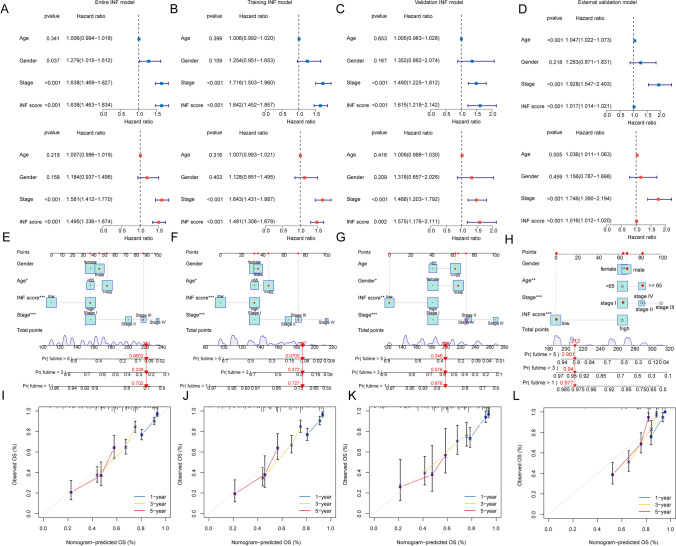
Independent prognostic analysis of the INF scoring system and construction of the nomogram diagnostic model. **A**–**D** Evaluation of the independent prognostic value of clinical-pathological features and INF scoring across four INF scoring models. **E**–**H** Construction of the nomogram diagnostic model based on clinical-pathological features and INF scoring. **I**–**L** Calibration curve analysis between the nomogram diagnostic model and actual survival probabilities

Integrating clinical-pathological variables of LUAD with the INF scoring system, we evaluated the independent prognostic value of each variable in predicting clinical outcomes of LUAD. In the entire INF scoring model, results from univariate and multivariate Cox analyses suggest that stage and the INF scoring system are independently associated with poor prognosis in LUAD (HR > 1, Fig. 7A). Both the training set and validation set of the INF scoring system model show consistent results in univariate and multivariate Cox analyses, indicating an association between INF score and poor prognosis in LUAD (HR > 1, Fig. 7B, C). In the external validation cohort, the results of univariate and multivariate Cox analyses indicated that age, stage, and INF score were associated with poor prognosis in LUAD (HR > 1, Fig. 7D).

Based on these findings, we conclude that the INF scoring system model, as an independent prognostic variable for LUAD, can accurately assess clinical survival outcomes. Furthermore, we conducted a comprehensive analysis of clinical variables and the INF scoring system model to construct a nomogram diagnostic model for predicting one-year, three-year, and five-year survival probabilities of LUAD samples in four different INF score models (Fig. 7E–H). Calibration curves evaluating the diagnostic accuracy of the nomogram diagnostic model show satisfactory consistency between predicted and actual survival probabilities across the four independent INF scoring models (Fig. 7G–L).

### Analysis of immune infiltration characteristics in INF scoring subgroups

**Fig. 8:**
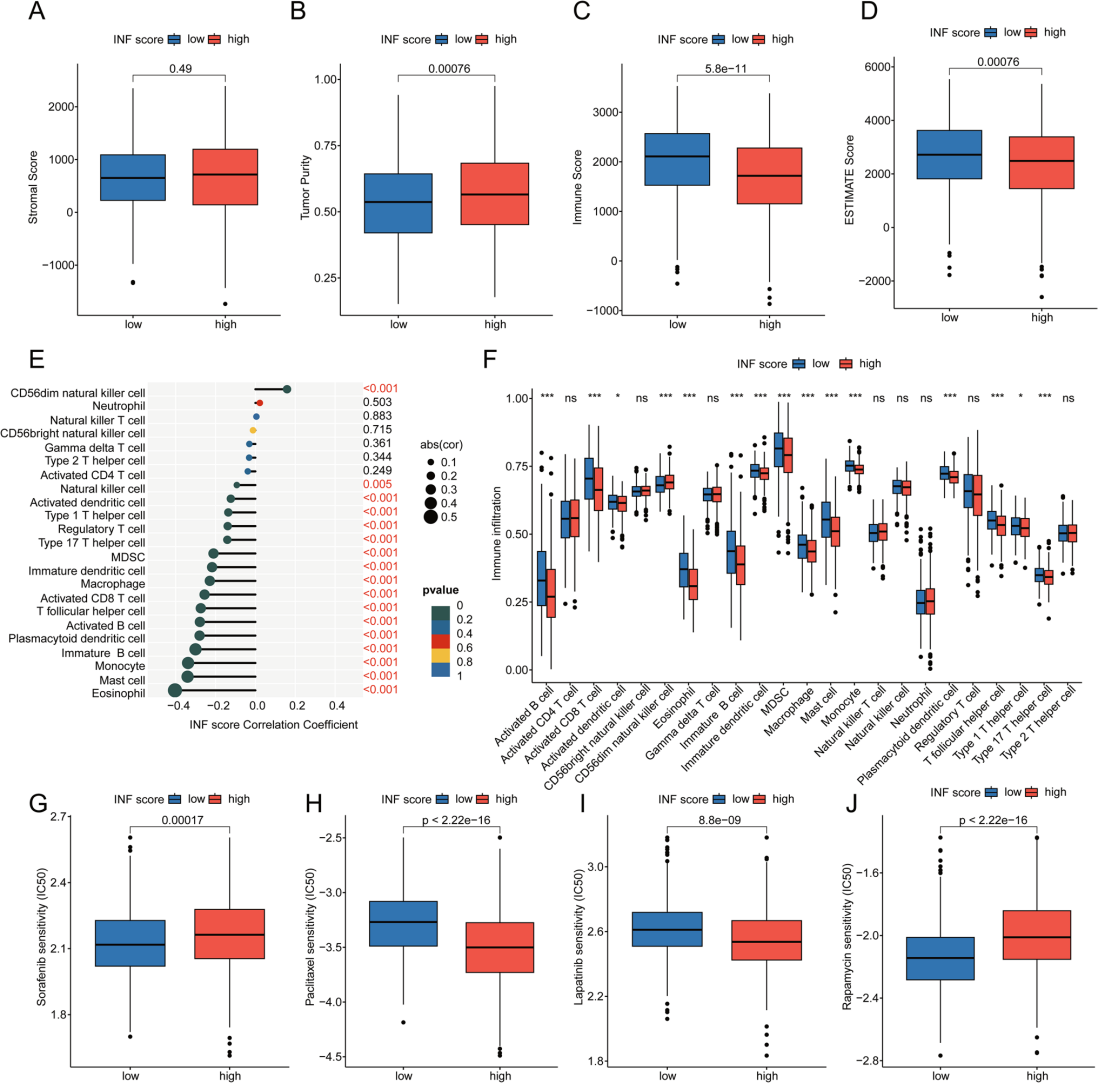
Assessment of immune infiltration characteristics and drug sensitivity analysis in INF scoring subgroups. **A**–**D** Evaluation of immune infiltration status based on ESTIMATE algorithm within INF scoring subgroups. **E** Correlation analysis between INF scoring and 23 immune cell infiltrations. **F** Analysis of immune cell infiltration proportions within INF scoring subgroups. **G**–**J** Drug sensitivity analysis

Using the ESTIMATE evaluation algorithm, we explored the immune infiltration status within INF scoring subgroups. Immune status analysis indicated higher tumor purity and significantly decreased immune and ESTIMATE scores in the high INF scoring subgroup compared to the low INF scoring subgroup, suggesting activated immune status may correlate with poor prognosis in LUAD (Fig. 8A–D). Using ssGSEA algorithm, we quantitatively analyzed the relative proportions of 23 immune cell infiltrations within INF scoring subgroups. Correlation analysis showed significant negative correlations between INF scoring and most immune cells, such as Eosinophil, Mast cell, Monocyte, Immature B cell, Plasmacytoid dendritic cell, and Activated B cell (Fig. 8E). Quantitative analysis revealed significantly reduced proportions of immune cell infiltrations in the high INF scoring subgroup, including Activated B cell, Activated CD8 T cell, Immature B cell, and Type 17 T helper cell (Fig. 8F). Analysis of drug sensitivity indicated lower IC50 scores for Sorafenib and Rapamycin in the low INF scoring subgroup, while higher IC50 scores were observed for Paclitaxel and Lapatinib compared to the high INF scoring subgroup (Fig. 8G–J).

### Analysis of somatic mutation characteristics and immune therapy response

**Fig. 9:**
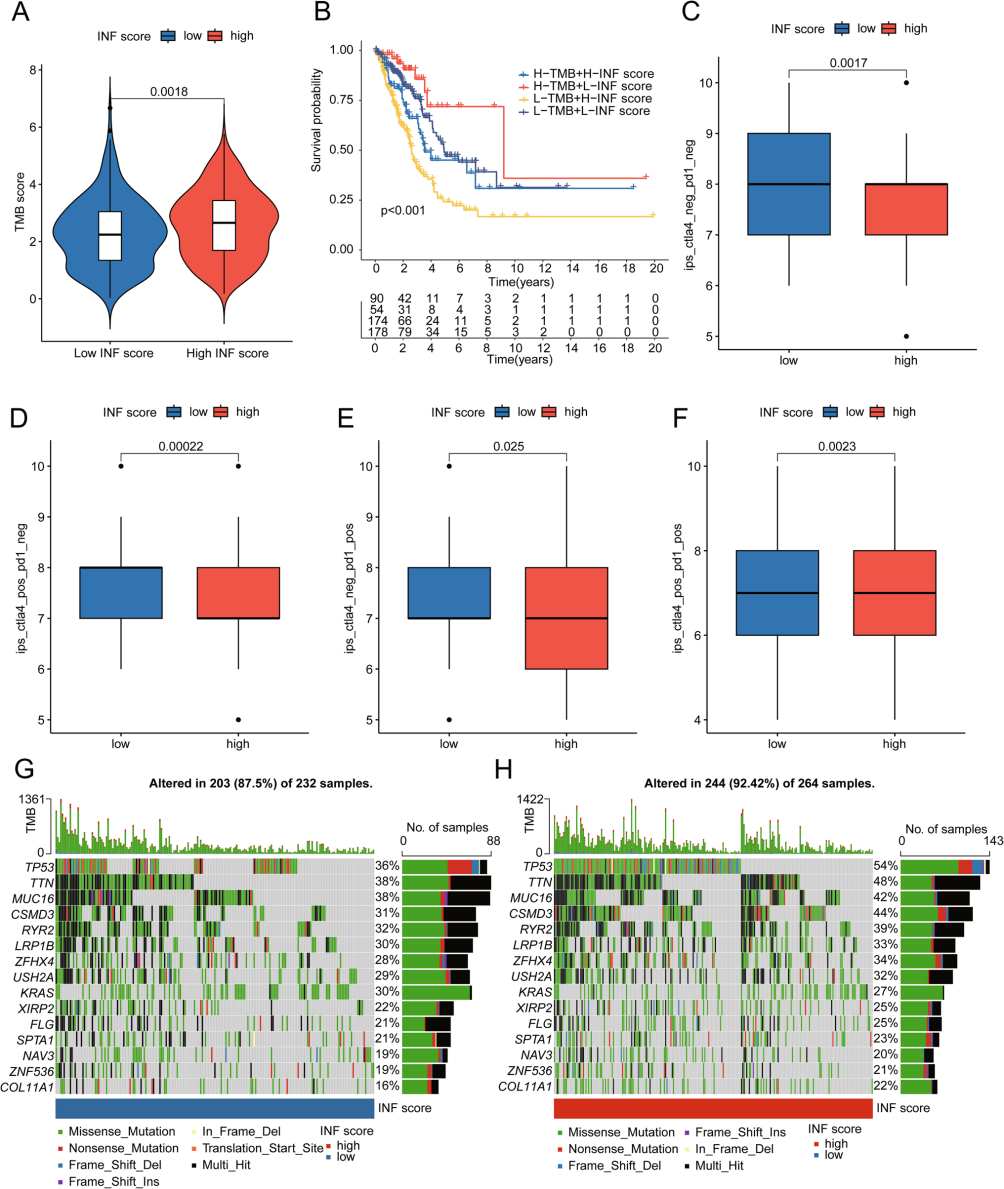
Somatic mutation characteristics and immune therapy response prediction in INF scoring subgroups. **A** Analysis of TMB scores in INF scoring subgroups. **B** Survival prognosis analysis in TMB mutation subgroups. **C**–**F** Prediction of IPS scores in INF scoring subgroups. **G**, **H** Analysis of somatic mutation characteristics in INF scoring subgroups

We further analyzed tumor mutation characteristics and immune therapy responses within INF scoring subgroups. Analysis of tumor mutation burden (TMB) showed a higher mutation burden in LUAD samples from the high INF scoring subgroup (Fig. 9A). Moreover, within TMB subgroups, clinical outcomes were better in the low INF scoring subgroup, whether in high or low TMB subgroups (Fig. 9B). Immune checkpoint inhibitor (ICI) Prediction Score (IPS) results revealed response patterns to PD-1 or CTLA-4 immune therapy in INF scoring subgroups, suggesting potentially better immune therapy responses in the low INF scoring subgroup (Fig. 9C–F). Somatic mutation analysis showed that among 232 samples in the low INF scoring subgroup, 203 samples exhibited somatic mutations (87.5%), whereas among 264 samples in the high INF scoring subgroup, 244 samples showed significant somatic mutations (92.42%). Notably, in the high INF scoring subgroup, mutations in TP53, TTN, MUC16, CSMD3, and RYR2 were observed at frequencies of 54%, 48%, 42%, 44%, and 39%, respectively, significantly higher than those in the low INF scoring subgroup (Fig. 9G, H). Based on these findings, we systematically discussed somatic mutation characteristics and potential immune therapy responses in INF scoring subgroups, providing new insights for future precision individualized therapy in LUAD.

### Downregulation of MMP14 can inhibit the cloning, proliferation, and invasion of lung cancer cell

**Fig. 10:**
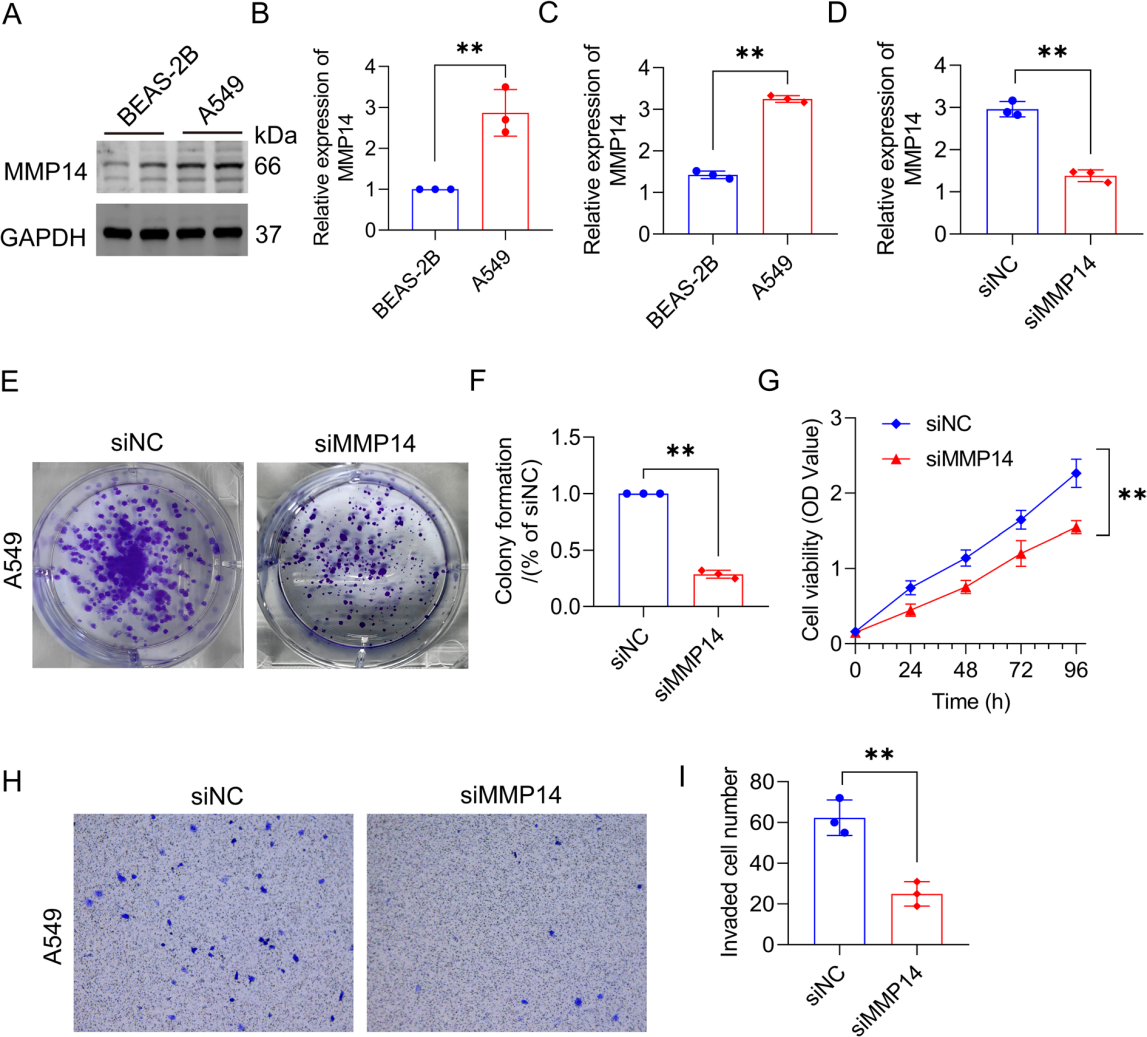
MMP14 regulated the proliferation and invasion of lung cancer cell. **A**, **B** MMP14 expression in BEAS-2B and A549 cells were analyzed by Western blot. **C** The levels of MMP14 were detected by qRT-PCR in BEAS-2B and A549 cells. **D** MMP14 expression in A549 cells was analyzed by qRT-PCR after treatment with MMP14 siRNA. **E**, **F** Colony formation assay was performed to detect the proliferation ability of cells after interfering with MMP14 siRNA. **G** MTT assay revealed that knockout of MMP14 reduced the growth rate. **H**, **I** Cell invasion ability was decreased when transfected with MMP14 siRNA. Cell invasion abilities were measured by a transwell assay method. *p < 0.05; **p < 0.01; ***p < 0.001

Western bolt and qRT-PCR were used to validate the expression of MMP14 in both lung cancer cell line A549 and normal bronchial epithelial cell line BEAS-2B. Results indicated a significant upregulation of MMP14 in lung cancer cells, which in line with the bioinformatics analysis findings (Fig. 10A–C). To examine the role of MMP14 in colony formation, proliferation and invasion, we transfected A549 cells with empty vector siNC or MMP14 siRNA (Fig. 10D). Colony formation assays revealed that MMP14 silencing decreased the mean colony number (Fig. 10E–F). The MTT assay demonstrated that MMP14 knockout markedly inhibited cell proliferation (Fig. 10G). Moreover, a Transwell assay showed that knockdown of MMP14 inhibited the invasion of cells compared with the cells transfected with inhibitor NC (Fig. 10H–I).

## Discussion

In this study, we provided a new risk stratification approach to inflammation and validated its reliability. Inflammation is an important effect indicator of immune response [16]. In the case of immune response dysfunction, combined with immune checkpoint pathway dysfunction, metabolic reprogramming, and cytokine dysregulation, tumors eventually show clinical symptoms [17–20]. Therefore, we further explored the importance of inflammatory process in LUAD.

Immunotherapy has been integrated into the treatment of early and localized non-small cell lung cancer [21–23]. Due to the difference in efficacy and common immunotoxicity, it is necessary to predict the effect of ICI and other immunotherapies [24]. However, how to accurately judge the effectiveness of immunotherapy is still an ongoing issue. Currently methods include PD-L1 expression, tumor mutation load (TMB), ctDNA, complete blood count, intestinal microbiome and other biomarker protocols [25–28]. We provide a scheme for risk stratification based on inflammation, resulting in differences in ICI sensitivity. Further understanding of the immunobiology of LUAD is critical to determine the role of the immune system in controlling tumor formation, progression, and metastasis [29].

Our clinical feature analysis based on INF score showed that in LUAD patients with early stage, the level of INF score had significant significance for the survival of patients, while in LUAD patients with stage III–IV, no difference in prognosis was observed in INF score among all groups. Surgery is the preferred treatment for patients with stage I and II of LUAD [30]. Our conclusions suggest the important value of inflammation in early LUAD patients and support perioperative immune-related treatment such as ICI, which is in line with previously observed conclusions [31, 32]. For inoperable early LUAD, radiation therapy changes the irradiated TME from immune resistance to immunogenicity, which is accompanied by an up-regulation of immune escape response [33]. Therefore, the combination of radiotherapy and ICI therapy has a theoretical basis and preliminary clinical effect [34, 35]. Our results suggest that early LUAD and advanced LUAD may have different inflammation status, which should be further explored in detail.

Eosinophils play an important role in regulating multiple processes including immune response, angiogenesis in TME [36]. Our results show that eosinophils content in TME of LUAD patients with a relatively good prognosis is significantly higher than that in the high-INF group with a relatively poor prognosis. A study of 158 patients with advanced LUAD showed that absolute eosinophil counts predicted clinical outcomes and toxicity in patients with LUAD receiving immunotherapy, and there was a significant association between higher eosinophil counts and better clinical outcomes [37]. Another study of 166 LUAD patients showed that eosinophil counts were associated with OS in patients treated with ICI and eosinophils therefore have the potential to serve as a new predictive biomarker [38]. Peripheral eosinophilic counts were associated with immune-related adverse events after ICI treatment and with improved clinical outcomes of immunotherapy in patients with advanced LUAD [39, 40]. The above results are in line with what we observe. In addition, eosinophilia is a biomarker for positive results after anti-PD-1 therapy in many tumors [36]. Elevated eosinophil count could be observed following pembrolizumab treatment for LUAD patients [41]. These evidences suggest that eosinophils are an important basis for the selection of immunotherapy for LUAD and an important indicator indicating the effectiveness of treatment.

Our results showed that the INF score had significant prognostic value for survival in stage I and II LUAD patients, while no significant prognostic differences were observed in stage III–IV patients. Although an exact explanation remains unclear, several possible clues can be considered from different perspectives. First, we found that only 180 LUAD samples were included in the stage III–IV group compared to other clinical subgroups, which may be an important reason for the lack of significant prognostic differences. Reports have indicated that early-stage LUAD patients often have surgical opportunities, whereas lobectomy in stage III-IV patients is not significantly associated with improved survival [42]. Whether INF classification is related to surgery and perioperative survival requires further exploration. Compared to stage III–IV patients, stage I and II patients exhibit higher immune and stromal scores [43]. Tumors in stage III–IV patients are often in an immunosuppressive state, and immune escape mechanisms in the tumor microenvironment may significantly affect the effectiveness of the INF score [44]. Resistance to chemotherapy or immunotherapy in stage III-IV patients may also limit the predictive capability of the scoring system in these patients [45]. Additionally, different genomic events between early- and late-stage LUAD patients may contribute to the heterogeneous predictive performance of the INF score [46, 47]. Therefore, although the INF score has prognostic value in early-stage LUAD, it may struggle to accurately reflect prognostic differences in immunosuppressive late-stage patients.

Our in vitro results showed that downregulation of MMP14 inhibited the cloning, proliferation and invasion of lung cancer cells, which is consistent with the previous observation that MMP14 promotes the migration and invasion of lung adenocarcinoma cells [48]. As a matrix metalloproteinase (MMP), MMP14 is involved in the degradation of various ECM components and processing and shedding membrane-bound proteins, which makes it capable of tumor-related functions [49]. MMP14 raised in several types of cancer, promote angiogenesis, inflammation, cancer cell invasion and metastasis [50–52]. In LUAD, the regulation of MMP14 at mRNA and protein levels can promote tumor progression and induce macrophage M2 polarization [53]. Concerning the impact of M2 macrophages on the development of LUAD, it may partially explain the mechanism of action of MMP14 on LUAD [54]. In addition, MMP14 can interact with bone sialoprotein to promote osteolytic bone metastasis in lung cancer [55]. Therefore, it is necessary to further explore the role and its clinical value as an intervention target of MMP14 in LUAD.

In summary, we provided a new risk stratification approach for inflammation in LUAD and partially validated its reliability. One of the weaknesses of this study is that we were unable to conduct a comprehensive analysis of how the cohort samples received immunotherapy based on public database data, limiting our ability to accurately study inflammation status and immunotherapy response. Although we provided simple in vitro experiments to validate the reliability of our bioinformatics results, this is far from sufficient for a comprehensive investigation of INF functions and mechanisms. Currently, we are conducting related mechanistic studies at both the cellular and animal levels to evaluate the immunotherapy response in LUAD patients and perform comparative analysis with our bioinformatics findings. Deep sequencing of LUAD samples using next-generation sequencing (NGS) technology is also necessary to comprehensively assess somatic mutation profiles and validate prognosis-related mutation types. Additionally, comparing the INF scoring system with other single-variable evaluation standards, such as TMB and PD-L1, for predicting clinical immunotherapy responses holds significant clinical value. In conclusion, further evaluation of the INF score system and the inflammatory process will help improve the efficacy of immune checkpoint inhibitors, providing more personalized and precise treatment for LUAD patients.

## Supplementary Information

Additional file1 (DOCX 240 KB)

## Data Availability

The data underpinning the findings of this study can be accessed by contacting the corresponding author with a reasonable request.
