# Supplementary material for: Molecular subtype characteristics and development of prognostic model based on inflammation-related gene in lung adenocarcinoma
Source: Discov Oncol. 2025 May 23;16:875. doi: 10.1007/s12672-025-02513-3 (PMC12102027; doi:10.1007/s12672-025-02513-3)
Supplement: Supplementary file 1 — Additional file1 (DOCX 240 KB) [file 12672_2025_2513_MOESM1_ESM.docx]

Supplementary Figure 1. Batch effect correction of transcriptome matrices for GSE72094 and TCGA databases. (A) PCA analysis before batch effect correction. (B) PCA analysis after batch effect correction.


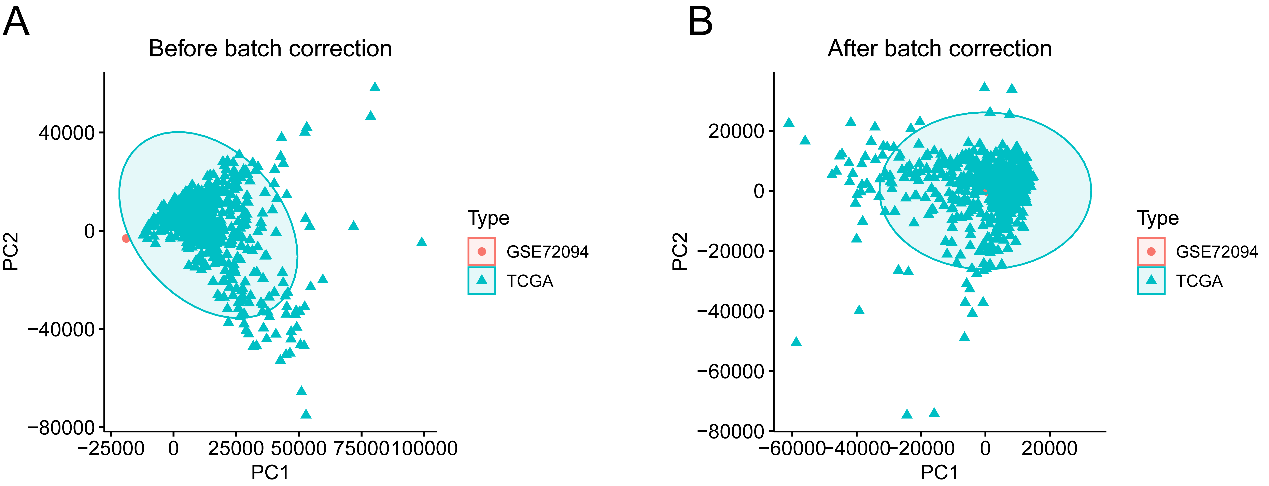


Supplementary Figure 2. Batch effect correction of transcriptome matrices for GSE31210 and GSE37745 datasets. (A) PCA analysis before batch effect correction. (B) PCA analysis after batch effect correction.


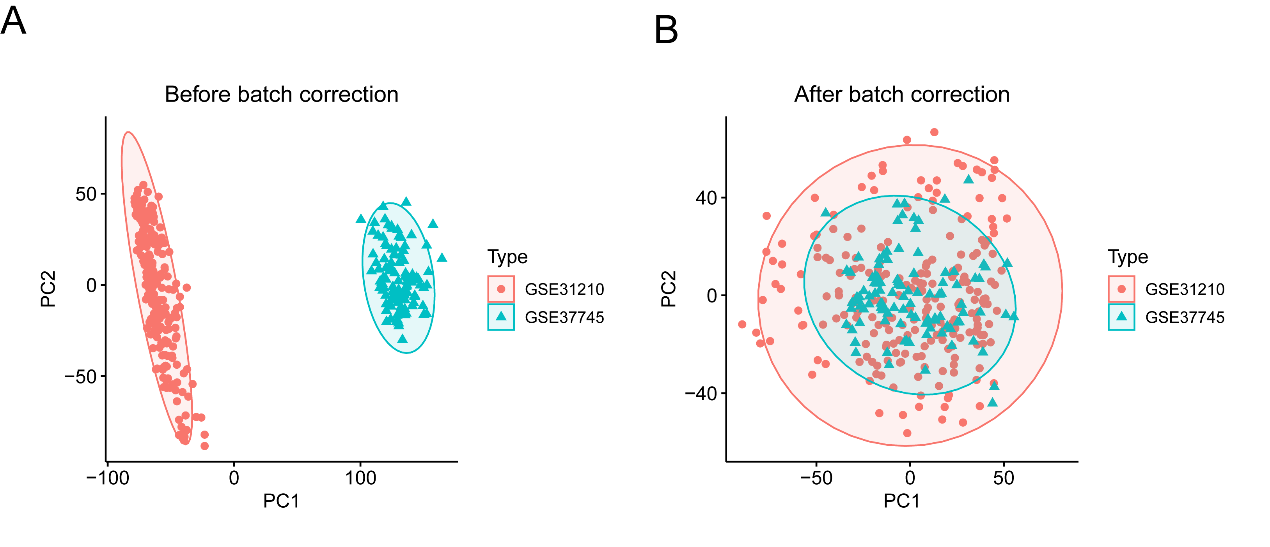


Supplementary Table 1. The risk coefficient of independent prognosis variable based on multivariate Cox analysis.

| Prognosis signature | Risk coefficient |
| --- | --- |
| MMP14 | 0.197416 |
| HPN | -0.06359 |
| BTG2 | -0.08629 |
| CCRL2 | -0.05132 |
| MET | 0.075936 |
| VIP | -0.10135 |
| TIMP1 | 0.131363 |
| GPC3 | -0.01117 |
| IL7R | -0.12932 |
| PCDH7 | 0.175565 |
| MEP1A | -0.23246 |
| OLR1 | -0.16976 |
| NMUR1 | -0.23128 |
